# Editorial: Integrating musculoskeletal ultrasound and emerging imaging modalities in surgical planning and rehabilitation

**DOI:** 10.3389/fsurg.2026.2004684

**Published:** 2026-10-15

**Authors:** Jaime Almazán-Polo, Ángel González-de-la-Flor, César Calvo-Lobo

**Affiliations:** 1 Faculty of Nursing, Physiotherapy and Podiatry, Department of Physiotherapy, Universidad Complutense de Madrid, Madrid, Spain; 2 Faculty of Sport Sciences, Universidad Europea de Madrid, Villaviciosa de Odón, Madrid, Spain

**Keywords:** advanced imaging analysis, artificial intelligence, imaging integration, musculoskeletal ultrasound (MSUS), quantitative ultrasound (QUS) image, shear wave elastography, surgical planning, ultrasound imaging and techniques

## Introduction and aims of the Research Topic

Musculoskeletal disorders represent a major and growing contributor to disability worldwide, with their burden expected to increase substantially over the coming decades (1). Within this context, advances in high-resolution ultrasound, shear wave elastography (SWE), magnetic resonance imaging (MRI), radiomics, and artificial intelligence are changing the role of imaging from anatomical description toward quantitative assessment, risk stratification, and clinical decision support.

This Research Topic aimed to explore how ultrasound and emerging imaging technologies can be integrated across the surgical and rehabilitation continuum: from diagnosis and preoperative planning to intraoperative risk assessment, postoperative monitoring, and evaluation of functional recovery. The six contributions illustrate this translational pathway through applications involving peripheral nerves, skeletal muscle, ligament and tendon injuries, fracture-related vascular risk, and artificial intelligence-assisted diagnosis.

## Quantitative and multimodal tissue assessment

Quantitative ultrasound provides accessible and radiation-free measurements of muscle architecture, echogenicity, tissue stiffness, and other potential imaging biomarkers, although acquisition and interpretation remain operator- and protocol-dependent, as discussed by Ashir et al. (2). This challenge is particularly relevant for SWE, for which probe orientation, anatomical location, tissue position, and acquisition settings can substantially influence the resulting values. Standardized examination procedures, such as those proposed by Zhu et al. (3), are therefore essential for improving reproducibility and clinical translation. Current evidence supports the diagnostic potential of SWE in musculoskeletal disorders, while also emphasizing methodological heterogeneity across studies, as summarized by Chowdhary et al. (4).

Within the Research Topic, Ma et al. evaluated muscle thickness, fiber length, and pennation angle of the tibialis anterior and medial gastrocnemius in patients after stroke. Their findings showed that ultrasound-derived muscle architecture was associated with rehabilitation outcomes and provided incremental predictive information beyond conventional functional scales. This application complements previous evidence on elastographic assessment of post-stroke muscle stiffness reported by Roots et al. (5) and illustrates how imaging biomarkers may contribute to individualized rehabilitation planning.

Peripheral nerve imaging further exemplifies the transition from structural assessment to quantitative and functionally meaningful biomarkers. Neto et al. (6) identified considerable variability in normative peripheral nerve SWE values associated with nerve location, limb position, transducer orientation, and acquisition protocols. Sánchez-Ramírez et al. (7) subsequently provided pooled evidence supporting the reliability of ultrasound and SWE measurements in lower-extremity nerves, particularly for sciatic nerve stiffness, while identifying more limited evidence for the femoral and saphenous nerves. Extending this methodological perspective, Cotteret et al. (8) explored the relationship between sciatic nerve stiffness and posterior-chain mobility. Although continuous correlations with flexibility tests were not significant, participants with limited mobility exhibited greater sciatic nerve stiffness.

In a direct diagnostic application, Yu et al. combined median nerve cross-sectional area adjusted for body surface area with Superb Microvascular Imaging and SWE for carpal tunnel syndrome. The multimodal model achieved better diagnostic performance than isolated morphological measurements, supporting the integration of anatomy, vascularity, and mechanical properties. These developments align with the broader potential of ultrasound in musculoskeletal rehabilitation described by Loomis et al. (9).

## Imaging informed surgical planning and safety

Advanced imaging can also improve surgical planning by identifying lesion characteristics, anatomical relationships, and patient-specific risks. Hattori et al. (10) emphasized the growing contribution of ultrasound-guided surgery and artificial intelligence to musculoskeletal care, while quantitative MRI provides complementary information concerning tissue composition, inflammation, degeneration, and biomechanics, as reviewed by Eck et al. (11).

Mei et al. developed an MRI-based classification system for Grade III medial collateral ligament injuries and compared imaging findings with intraoperative observations in 226 cases. The system demonstrated high diagnostic accuracy for localizing ligament tears and emphasized the clinical relevance of posterior oblique ligament involvement when considering acute repair. This contribution illustrates how structured interpretation of preoperative imaging can translate anatomical findings into more precise surgical decisions.

The case reported by Zhang et al. provides an important counterpoint to an exclusively technology-driven interpretation of imaging. Preoperative CT demonstrated a potentially hazardous anatomical relationship between the displaced lesser trochanter fragment and the profunda femoris vein, whereas Doppler ultrasound did not reveal vascular injury. Nevertheless, a venous laceration was identified during surgical exploration. Rather than representing an isolated failure of a particular modality, this discrepancy illustrates that imaging findings must be interpreted within the broader clinical context, considering fracture morphology, injury mechanisms, anatomical risk, and the level of clinical suspicion. Imaging techniques, predictive models, and diagnostic algorithms should therefore be understood as decision-support tools that complement—but do not replace—clinical examination, professional judgement, and, when indicated, direct surgical assessment. A negative imaging result should not automatically exclude a clinically plausible complication, particularly when other anatomical or clinical findings suggest an increased risk.

## Prediction, radiomics, and artificial intelligence

The transition from visualization to prediction is illustrated by Liao et al., who combined MRI radiomics features with clinical variables in a nomogram predicting functional outcomes after rotator cuff repair. The model integrated age, pain, tear morphology, tendon retraction, and quantitative image features, illustrating the potential of imaging to support individualized prognosis.

Similarly, Wang et al. developed a 2.5-dimensional deep learning and ensemble-learning framework for detecting superior labrum anterior-to-posterior lesions on MRI. The model showed favorable sensitivity and anatomically coherent attention to the biceps–labral complex. These findings reflect the expanding applications of artificial intelligence in musculoskeletal imaging described by Gitto et al. (12) and in related areas such as spinal imaging by Lee et al. (13).

However, clinical translation requires transparent reporting, external validation, representative datasets, and reproducible image processing. Standardization initiatives such as that reported by Whybra et al. (14) are essential for reducing variability in radiomic feature extraction. The reproducibility and validation limitations identified by Gitto et al. (15) in musculoskeletal tumor radiomics are equally relevant to predictive models across orthopedics and rehabilitation.

## Summary and future directions

Together, the contributions to this Research Topic demonstrate that imaging is evolving from a primarily diagnostic resource into a longitudinal and integrative component of patient care. Ultrasound and MRI can characterize not only tissue structure but also its mechanical and physical properties, providing quantitative information about tissue behavior and functional status. These techniques can also help anticipate surgical risks, support patient-specific management, and provide biomarkers associated with tissue healing and functional recovery.

Nevertheless, imaging findings and derived tools, including radiomics, artificial intelligence, and machine-learning models, must remain subordinate to clinical reasoning and direct patient examination. They should complement the diagnostic process and support decision-making but should not, in isolation, determine clinical, rehabilitative, or surgical decisions. A negative imaging result should not automatically exclude a clinically plausible condition, particularly when anatomical findings, injury mechanisms, or clinical examination indicate otherwise.

Future research should prioritize standardized acquisition protocols, reproducible measurements, multicenter validation, clinically interpretable models, and the integration of imaging biomarkers with meaningful patient outcomes. Close collaboration among surgeons, radiologists, physiotherapists, engineers, and data scientists will be fundamental to translating these advances into safer surgery and more personalized rehabilitation. The future of musculoskeletal imaging will therefore depend not only on technological sophistication, but also on its responsible integration into clinically grounded diagnostic and therapeutic pathways.

## References

[1] Gill TK Mittinty MM March LM Steinmetz JD Culbreth GT Cross M. Global, regional, and national burden of other musculoskeletal disorders, 1990–2020, and projections to 2050: a systematic analysis of the global burden of disease study 2021. Lancet Rheumatol. (2023) 5:e670–82. 10.1016/S2665-9913(23)00232-1 37927903 PMC10620749

[2] Ashir A Jerban S Barrère V Wu Y Shah SB Andre MP. Skeletal muscle assessment using quantitative ultrasound: a narrative review. Sensors (Basel). (2023) 23. 10.3390/S23104763 37430678 PMC10222479

[3] Zhu J Qiu L Ta D Hua X Liu H Zhang H. Chinese ultrasound doctors association guideline on operational standards for 2-D shear wave elastography examination of musculoskeletal tissues. Ultrasound Med Biol. (2024) 50:175–83. 10.1016/j.ultrasmedbio.2023.10.005 37949764

[4] Chowdhary K Raum G Visco C. Diagnostic utility of shear wave elastography in musculoskeletal injuries: a narrative review. PM R. (2024) 16:384–97. 10.1002/PMRJ.13179 38607311

[5] Roots J Trajano GS Fontanarosa D. Ultrasound elastography in the assessment of post-stroke muscle stiffness: a systematic review. Insights Imaging. (2022) 13. 10.1186/S13244-022-01191-X 35380302 PMC8982789

[6] Neto T Johannsson J Andrade RJ. Using ultrasound shear wave elastography to characterize peripheral nerve mechanics: a systematic review on the normative reference values in healthy individuals. Ultrasonography. (2024) 43:169–78. 10.14366/USG.23211 38544459 PMC11079506

[7] Sánchez-Ramírez O Almazán-Polo J González-de-la-Flor Á. Reliability of ultrasound and shear wave elastography in assessing lower extremity nerve stiffness and excursion: a systematic review and meta-analysis. Ultrasound Med Biol. (2025) 51:1043–58. 10.1016/j.ultrasmedbio.2025.03.006 40251089

[8] Cotteret C Almazán-Polo J González-de-la-Flor Á. Shear wave elastography of the sciatic nerve and its relationship with posterior chain flexibility in healthy participants: an observational study. Sensors (Basel). (2025) 25. 10.3390/S25092885 40363321 PMC12074453

[9] Loomis KJ Shin J Roll SC. Current and future utility of ultrasound imaging in upper extremity musculoskeletal rehabilitation: a scoping review. J Hand Ther. (2024) 37:331–47. 10.1016/j.jht.2023.09.014 37863730

[10] Hattori S Saggar R Heidinger E Qi A Mullen J Fee B. Advances in ultrasound-guided surgery and artificial intelligence applications in musculoskeletal diseases. Diagnostics (Basel). (2024) 14. 10.3390/DIAGNOSTICS14182008 39335687 PMC11431371

[11] Eck BL Yang M Elias JJ Winalski CS Altahawi F Subhas N. Quantitative MRI for evaluation of musculoskeletal disease: cartilage and muscle composition, joint inflammation, and biomechanics in osteoarthritis. Invest Radiol. (2023) 58:60–75. 10.1097/RLI.0000000000000909 36165880 PMC10198374

[12] Gitto S Serpi F Albano D Risoleo G Fusco S Messina C. AI applications in musculoskeletal imaging: a narrative review. Eur Radiol Exp. (2024) 8. 10.1186/S41747-024-00422-8 PMC10866817 38355767

[13] Lee S Jung JY Mahatthanatrakul A Kim JS. Artificial intelligence in spinal imaging and patient care: a review of recent advances. Neurospine. (2024) 21:474–86. 10.14245/NS.2448388.194 38955525 PMC11224760

[14] Whybra P Zwanenburg A Andrearczyk V Schaer R Apte AP Ayotte A. The image biomarker standardization initiative: standardized convolutional filters for reproducible radiomics and enhanced clinical insights. Radiology. (2024) 310(2):e231319. 10.1148/RADIOL.231319 PMID: 38319168; PMCID: PMC10902595. 38319168 PMC10902595

[15] Gitto S Cuocolo R Huisman M Messina C Albano D Omoumi P. CT and MRI radiomics of bone and soft-tissue sarcomas: an updated systematic review of reproducibility and validation strategies. Insights Imaging. (2024) 15. 10.1186/S13244-024-01614-X PMC10899555 38411750

